# *In Vitro* Ion Chelating, Antioxidative Mechanism of Extracts from Fruits and Barks of *Tetrapleura tetraptera* and Their Protective Effects against Fenton Mediated Toxicity of Metal Ions on Liver Homogenates

**DOI:** 10.1155/2015/423689

**Published:** 2015-08-18

**Authors:** Bruno Moukette Moukette, Anatole Constant Pieme, Prosper Cabral Nya Biapa, Jacques Romain Njimou, Marco Stoller, Marco Bravi, Jeanne Yonkeu Ngogang

**Affiliations:** ^1^Laboratory of Biochemistry, Department of Biochemistry and Physiological Sciences, Faculty of Medicine and Biomedical Sciences, University of Yaoundé I, P.O. Box 1364, Yaounde, Cameroon; ^2^Department of Biochemistry, Faculty of Science, University of Dschang, P.O. Box 67, Dschang, Cameroon; ^3^Department of Chemical Materials Environmental Engineering, University of Rome “La Sapienza”, Via Eudossiana No. 18, Piazzale Aldo Moro 5, 00185 Rome, Italy

## Abstract

The aim of the present study was to investigate the antioxidant activity and protective potential of *T. tetraptera* extracts against ion toxicity. The antioxidant activity of the extracts was investigated spectrophotometrically against several radicals (1,1-diphenyl-2-picrylhydrazyl (DPPH^•^), 2,2′-azino-bis(3-ethylbenzthiazoline-6-sulfonic acid) (ABTS^•^), hydroxyl radical (HO^•^), and nitric oxide (NO^•^)), followed by the ferric reducing power, total phenols, flavonoid, and flavonol contents. The effects of the extracts on catalase (CAT), superoxide dismutase (SOD), and peroxidase activities were also determined using the standard methods as well as the polyphenol profile using HPLC. The results showed that the hydroethanolic extract of *T. tetraptera* (CFH) has the lowest IC_50_ value with the DPPH, ABTS, OH, and NO radicals. The same extract also exhibited the significantly higher level of total phenols (37.24 ± 2.00 CAE/g dried extract); flavonoids (11.36 ± 1.88 QE/g dried extract); and flavonols contents (3.95 ± 0.39 QE/g dried extract). The HPLC profile of *T. tetraptera* revealed that eugenol (958.81 ± 00 mg/g DW), quercetin (353.78 ± 00 mg/g DW), and rutin (210.54 ± 00 mg/g DW) were higher in the fruit than the bark extracts. In conclusion, extracts from *T. tetraptera* may act as a protector against oxidative mediated ion toxicity.

## 1. Introduction

Several fundamental life functions such as enzymatic catalyzed reactions heavily rely on metal ions [1]. Metal ions are indispensable for cells metabolism in trace; however, in a higher quantity, they could be toxic for human. Chronic exposure to toxic metals leads to their accumulation in the living system and becomes a health threat. A common mechanism of toxicity involves redox-active metals via the generation of reactive oxygen species (ROS) through a Fenton reaction [1]. The classic Fenton process begins with the production of short-lived, extremely reactive hydroxyl radical (^•^OH) with a potential of 2.8 V from the interaction between Fe^2+^ and H_2_O_2_. A molecule of Fe^3+^ can also initiate a Fenton-like reaction even though the reduction of Fe^3+^ to Fe^2+^ occurs much more slowly than the backward process [2]. The formed ^•^OH then attacks the substrate, which results in substrate decomposition and production of more ROS. These species are highly reactive and short-lived because they possess one or more unpaired electrons, an unpaired electron being one that is alone in an orbital. The ROS create a disequilibrium state in the cell named oxidative stress (OS) which is a cellular redox imbalance in favor of prooxidant [3, 4]. The OS in his turn causes the damage of proteins, the peroxidation of lipids, and the alteration of DNA [5].

The redox state of essential transition metals such as Fe (II/III) makes them biologically important and also toxic during an OS process. The iron (Fe) in a healthy human body is present mostly in hemoglobin. It serves as essential cofactors for a multitude of enzymes, signaling molecules and regulates various biological pathways, including mitochondrial energy production, DNA replication/transcription, and synthesis of neurotransmitters. In the presence of hydrogen peroxide, these iron ions generate toxic hydroxyl radicals via a Fenton or Fenton-like reactions [5]. Redox-active iron (Fe) induces both genome damage and inhibits key enzymes in the oxidative genome damage machinery by altering their structure and reversibly oxidizing cysteine residues in these proteins [6].

In a previous study, the injection of FeCl_3_ into the* substantia nigra* of adult rats resulted in a substantial selective decrease of striatal dopamine (95%), leading to Parkinson's disease-like symptoms [7]. Metals have therefore been implicated in multiple pathologies, Alzheimer's disease, Parkinson's disease, and cancer [1].

In order to fight OS and oxidative mediated damage the human system has endogenous antioxidants (molecules capable of stabilizing or deactivating free radicals before they attack cells) which play a crucial role in maintaining optimal cellular functions, systemic health, and well-being [8, 9]. Indeed, humans have developed very complex antioxidant systems both enzymatic (glutathione peroxidase, catalase, and superoxide dismutase) and nonenzymatic (vitamins E and C, thiol antioxidants), which work in synergistic way to protect the cells and organ systems against free radical damage by quenching free radicals and/or chelating redox metals at physiologically related levels [9, 10]. However, under conditions which promote a high state of OS, endogenous antioxidants may not be sufficient [3, 11].

Nutritional therapy and phytotherapy have emerged as new concepts of health aid in recent years. Strong recommendations for consumption of nutraceuticals from plant origin have become progressively popular to improve health and to prevent and treat diseases [12]. Nutraceuticals are naturally derived bioactive compounds that are found in foods, dietary supplements, and herbal products and have health promoting, disease preventing, and medicinal properties [4, 13]. Food components have been for long studied for their biological activities including antioxidative and protective potential against ion-oxidative mediated toxicity [3, 14–17].* Tetrapleura tetraptera* (*T. tetraptera*), named “sassas” in* Bassa*; “essissa” in* Ewondo* is a leguminous of 25 m of height deciduous plant belonging to the Mimosaceae family. It is generally found in the lowland forest of tropic al Africa. The fruits consist of a fleshy pulp with small, brownish-black seeds. The fruits are green when tender and dark brown when fully ripe and possess high nutritional value [18]. The dry fruit has a pleasant aroma and hence they are used as spice in Central and West Africa. Plant derived functional foods have received considerable attention because of their presumed safety and potential nutritional and therapeutic effects [12]. In Africa,* T. tetraptera* is used in ethnomedicine for the treatment of several pathologies [18]. Previous studies have demonstrated the benefic effect on the treatment of* T. tetraptera* on intestinal parasites, diabetes mellitus, hypertension, arthritis, asthma, epilepsy, schistosomiasis, malaria, wound healing, and for prevention of postpartum contraction [19–21]. The fruit is also used as a dietary supplement rich in vitamins. It has been reported that* T. tetraptera* has the potential for antimicrobial activities [22]. Although these scientific studies provide many information on the biological properties of* T. tetraptera,* its protective properties against metal toxicity have not been studied. Therefore, the present study aims at determining the protective effect of* T. tetraptera* against ion mediated toxicity on rat liver* in vitro* together with its antioxidant properties.

## 2. Material and Methods

### 2.1. Plant Material

The fruits and barks of* T. tetraptera* were collected at the Kala Mountain in the Center region of Cameroon. They were authenticated by Mr. Nana, a botanist of the National Herbarium of Cameroon, in comparison to the voucher specimens (N. 1858/SRF).

### 2.2. Preparation of Plant Extracts

The collected fruits and barks were dried at ambient temperature and crushed. The powders were then macerated at the ratio of 1 : 10 (w/v) for 48 h in ethanol for the ethanolic extract and in a mixture of water/ethanol (30/70; pH = 3) for the hydroethanolic extract. The mixtures were then filtered using a Buchner funnel and Whatman number 1 filter paper. This process was repeated once on the residue. The supernatant was concentrated using a rotavapor and the extract was dried in the oven at 55°C for two days. Each crude extract obtained was labelled using the following codes: CEH:* T. tetraptera* hydroethanolic extract (bark); CFH:* T. tetraptera* hydroethanolic extract (fruit); CEE:* T. tetraptera* ethanolic extract (bark); CFE:* T. tetraptera* ethanolic extract (fruit). The different samples were then kept at 4°C. Prior to the experimentation, the solutions of the four plant extracts were dissolved using ethanol different dilutions (25, 50, 75, 150, and 300 *μ*g/mL) of each.

### 2.3. Animals

Male albino* Wistar* rats weighing 200–250 g were used in this study. The rats were maintained at room temperature under the lab conditions and were fed with standard diet and water* ad libitum*. Livers were collected after decapitation of the rats under mild ether anesthesia from overnight fasted rats. This study was carried out with approval from the Animal Ethics Committee of University of Yaoundé I.

### 2.4. Scavenging Activity of DPPH Radical

The DPPH assay measures the free radical scavenging capacity of the extracts as described previously [23]. Three milliliters of each of the diluted extracts was put in the test tube and 1 mL of a methanol solution of DPPH (0.1 mM) was added. The mixture was kept in the dark at room temperature for 30 min and absorbance was measured at 517 nm against a blank. The same procedure was used for the vitamin C used as standard. The following equation was used to determine the percentage of the radical scavenging activity of each extract:

(1) $$\begin{array}{c} \text{Scavenging effect }\left(\;\%\;\right)=100\times \frac{\left(A_{o}-A_{s}\right)}{A_{o}}, \end{array}$$

where *A*_*o*_ is the absorbance of the blank and *A*_*s*_ the absorbance of the sample.

### 2.5. Scavenging Effect of the ABTS^+^ Radical

The ABTS assay was based on a previously described method [24] with slight modifications. ABTS radical cation (ABTS^+^) was produced by the reaction of a 7 mM ABTS solution with potassium persulfate (2.45 mM). The ABTS^+^ solution was diluted with ethanol to an absorbance of 0.70 ± 0.05 at 734 nm. The mixture was stored in the dark at room temperature for 12 h before used. After addition of 25 *μ*L of extract sample or vitamin C used as standard to 2 mL of diluted ABTS^+^ solution, absorbance was measured at 734 nm after exactly 6 min. The decrease in absorption was used for calculating scavenging effect values. Equation (2) was used to determine the percentage of the radical scavenging activity of each extract.

### 2.6. Nitric Oxide Scavenging Activity

Nitric oxide scavenging activity was determined according to previous authors [25]. The reaction mixture contained 2 mL of sodium nitroprusside (10 mM) in 0.5 mL phosphate buffer (0.5 M; pH 7.4). Various concentrations (25, 50, 75, 150, and 300 *μ*g/mL) of the extracts (0.5 mL) were added in a final volume of 3 mL. After incubation for 60 min at 37°C, Griess reagent (*α*-naphthyl-ethylenediamine (0.1%) and sulphanilic acid (1%) in H_3_PO_4_ (5%)) was added. The pink chromophore generated during diazotization of nitrite ions with sulphanilamide and subsequent coupling with *α*-naphthyl-ethylenediamine was measured spectrophotometrically at 540 nm. Ascorbic acid was used as a positive control. The scavenging ability (%) of the nitric oxide was calculated using equation (2).

### 2.7. Hydroxyl Radical Scavenging Activity

The scavenging activity of the extracts on hydroxyl radical was measured according to a previously described method [26]. To 1.5 mL of each diluted extract, 60 *μ*L of FeCl_3_ (1 mM), 90 *μ*L of 1,10-phenanthroline (1 mM), 2.4 mL of phosphate buffer (0.2 M; pH 7.8), and 150 *μ*L of H_2_O_2_ (0.17 M) were added, respectively. The mixture was then homogenized using a vortex and incubated at room temperature for 5 min. The absorbance was read at 560 nm against the blank. The percentage of the radical scavenging activity of each extract was calculated from (2).

### 2.8. Total Antioxidant Activity by Ferric Reducing Antioxidant Power Assay

The FRAP was determined using a previously described method [27] with slight modifications. The fresh FRAP reagent consisted of 500 mL of acetate buffer (300 mM; pH 3.6), 50 mL of 2,4,6-Tri (2-pyridyl)-s-triazin (TPTZ) (10 mM), and 50 mL of FeCl_3_·6H_2_O (50 mM). The colorimetric measurement was performed at 593 nm and the measurement was monitored up to 12 min on 75 *μ*L of each extract and 2 mL of FRAP reagent. The vitamin C was used to draw a standard curve and the butylated hydroxytoluene (BHT) was used for the comparison. The results were expressed as mg equivalent vitamin C/g of dried extract (mg eq VitC/g DE).

### 2.9. Phosphomolybdenum Antioxidative Power (PAP)

The total antioxidant activity of the extracts was evaluated by green phosphomolybdenum complex [28]. An aliquot of 10 *μ*L of the extract solution was mixed with 1 mL of reagent solution (0.6 M sulphuric acid, 28 mM sodium phosphate, and 4 mM ammonium molybdate) in a microcentrifuge tube. The tubes were incubated in a dry thermal bath at 95°C for 90 min. After cooling, the absorbance of the mixture was measured at 695 nm against a blank. The vitamin C was used as reference to draw the standard curve and BHT was used for the comparison. The reducing capacities of the analysed extracts were expressed as mg of ascorbic acid equivalents/g of dried extract (mg eq AS/g DE).

### 2.10. Reducing Power Assay

The reducing power of the extracts was determined by a method described by Oyaizu [29]. Different concentrations of extracts in 1 mL of distilled water were mixed with 2.5 mL of phosphate buffer (0.2 M, pH 6.6) and 2.5 mL of potassium ferrocyanide (1%). The mixtures were incubated at 50°C for 20 min. Aliquots 2.5 mL of trichloroacetic acid (10%) were added to the mixtures and centrifuged at 3000 rpm for 10 min. The supernatant of the solution (2.5 mL) was mixed with 2.5 mL of distilled water and 0.5 mL of FeCl_3_ (0.1%). The absorbance was measured at 700 nm.

### 2.11. Total Phenol Determination

The total phenol content was determined by the Folin-Ciocalteu method [30]. The reaction mixture contained 200 *μ*L of extract, 800 *μ*L of freshly prepared diluted Folin-Ciocalteu reagent and 2 mL of sodium carbonate (7.5%). The final mixture was diluted to 7 mL with deionized water and kept in the dark at ambient conditions for 2 h to complete the reaction. The absorbance was measured at 765 nm. Caffeic acid was used as standard and the results were expressed as mg caffeic acid/g dried extract (mg CA/g DE).

### 2.12. Determination of Total Flavonoid Content

Total flavonoid content was determined using aluminium chloride (AlCl_3_) according to a known method [31] using quercetin as a standard. A volume of 0.1 mL of spice extract was added to 0.3 mL distilled water followed by 0.03 mL of NaNO_2_ (5%). After 5 min at 25°C, 0.03 mL of AlCl_3_ (10%) was added. After further 5 min, the reaction mixture was mixed with 0.2 mL of 1 mM NaOH. Finally, the reaction mixture was diluted to 1 mL with water and the absorbance was measured at 510 nm. The results were expressed as quercetin equivalent mg/g of dried extract (QE/g dried ext).

### 2.13. Determination of Total Flavonols

Total flavonols in the plant extracts were estimated using a known method [32] with modifications. To 2.0 mL of sample, 2.0 mL of 2% of ethanolic solution of AlCl_3_ and 3.0 mL (50 g/L) sodium acetate solutions were added. After 2.5 h of incubation at 20°C, the absorbance was read at 440 nm. The results were expressed as quercetin equivalent (mg/g) dried extract (QE/g dried ext).

### 2.14. Quantification of Phenolic Compounds by High Performance Liquid Chromatography (HPLC)

High Performance Liquid Chromatography (HPLC) with UV detection is frequently used for the separation and detection of phenolic compounds in extracts. Samples were dissolved in pure water according to the ratio (0.3 g/10 mL) and centrifuged at 4706 rpm for 10 min. The supernatant was filtered through a cellulose acetate membrane filter (0.20 *μ*m or 0.45 *μ*m, Schleicher and Schuell) and used for analysis. The analysis was performed on an Agilent Technologies 1200 HPLC system fitted with a SUPELCOSIL LC-18 column (length 250 mm, diameter 4.6 mm, and packaging size 5 mm). The column temperature was set at 20°C. The mobile phase consisted of a mixture of an aqueous solution of acetic acid (0.5%) by volume (“A”) and acetic nitrile (“B”). Elution was performed by using 100% of A for the first 2 min of the run, 40% of A and, 60% of B from 2 to 60 min. The flow rate was set equal to 1 mL/min and the injection volume was 25 microlitres. Polyphenols were detected by a UV detector (280 nm). The retention times of the identified polyphenolic compounds of interest were measured by a single standard solution at a concentration of 100 mg/L.

### 2.15. Protective Properties of the Plant against Oxidative Damage

#### 2.15.1. Preparation of Liver Homogenate

The liver was isolated from 3 normal albino* Wistar* rats. The organs were weighed and 10% (w/v) homogenate was prepared in phosphate buffer (0.1 M, pH 7.4 having 0.15 M KCl) using the homogenizer at 4°C [33]. The homogenate was centrifuged at 3000 rpm for 15 min and the clear cell-free supernatant obtained was used for the study.

#### 2.15.2. Preparation of the Prooxidative Solution

The oxidant solution was prepared, directly before its utilization by adding a solution of ferric chloride 100 mM to H_2_O_2_ 0.50% prepared in phosphate buffer (0.1 M, pH 7.4). This solution was used for the investigation of the protective assays on liver enzymes.

#### 2.15.3. Total Protein Content

The total protein content of the mixture of liver was measured according to the protein kit supplier method (Human Kit, Wiesbaden, Germany). This result was used to express the activities of the different enzymes per gram of organs.

#### 2.15.4. *In Vitro* Lipid Peroxidation Assay

Lipid peroxidation assay was performed by a formerly described protocol [34]. Phosphate buffer 0.58 mL (0.1 M; PH 7.4), 200 *μ*L sample, 200 *μ*L liver homogenate, and 20 *μ*L ferric chloride (100 mM) were combined to form mixture which was placed in a shaking water bath for 1 h at 37°C. The reaction was terminated by adding 1 mL TCA (10%), TBA 1 mL (0.67%) to all the tubes which were placed in boiling water bath for 20 min. Then test tubes were shifted to crushed ice bath and were centrifuged at 3000 trs/rpm for 10 min. Absorbance of the supernatant was checked at 535 nm and was calculated as nM of MDA tissue using molar extinction coefficient of 1.56 × 10^5^/M·cm.

#### 2.15.5. Determination of Peroxidase Activity

Peroxidase activity was determined by the peroxidase kit (CAS Number 7722-84-1, Sigma Aldrich) supplier with modifications. A solution containing the mixture of 1 mL of the oxidant solution (FeCl_3_, 100 mM) and extract or vitamin C (standard) for a final concentration of 100 *μ*g/mL was incubated for 1 h in a water bath at 37°C. An aliquot of PBS (0.1 mL), hydrogen peroxide (50 *μ*L), and pyrogallol solution (110 *μ*L) were added to distilled water (625 *μ*L) that was earlier dispensed into an Eppendorf tube. The plant extract (75 *μ*L) from the mixture was thereafter added. For the blank, the control oxidant solution and the vitamin C as standard, the same reagents were used, except the extract which was replaced by distilled water (75 *μ*L). The reaction was mixed and incubated for at least 10 min. The solution containing 100 mM, pH 6.0 PBS (40 *μ*L), and 0.002% (v/v) diluted liver homogenate (40 *μ*L) was added to the blank and test mixtures, respectively. These were mixed, and the increase in absorbance at 420 nm was measured at every 10 s for 3 min using a spectrophotometer (BioMate 3S UV-Visible, Thermo Scientific Manufacturer, Wohlen, Switzerland). One unit of peroxidase was defined as the change in absorbance/seconds/mg of protein at 420 nm using molar extinction coefficient of 12/M·cm.

#### 2.15.6. Determination of Catalase Activity

Prior to the test, a solution containing a mixture of 1 mL of total volume of the oxidant solution and extract or vitamin C (standard) for a final concentration of 100 *μ*g/mL was incubated for 1 h in a water bath at 37°C. The catalase activity of liver homogenate was assayed as previously described with modifications [35]. An aliquot of hydrogen peroxide (0.8 mL) was dispensed into an Eppendorf tube. Phosphate buffer (1.0 mL), extracted sample/vitamin C/oxidant solution (75 *μ*L) and (0.002% v/v) diluted homogenate (125 *μ*L) were added. The reaction mixture (0.5 mL) was dispensed into 5% dichromate reagent (1.0 mL) and vigorously shaken. The mixture was heated in a Clifton water bath for 10 min and allowed to cool. The absorbance at 570 nm was taken using spectrophotometer (BioMate 3S UV-Visible, Thermo Scientific Manufacturer, Wohlen, Switzerland). The absorbance obtained was extrapolated from the following standard curve *y* = 0.0028*x* + 0.0132. The catalase activity was thereafter expressed as unit/min/mg of protein (UI/mg Prot.):

(2) $$\begin{array}{c} \mathrm{CAT}\left(\;\frac{\left(\text{unit}\right)}{\text{mg protein}}\;\right)=\frac{\left(\text{Abs}/\min\times 30000\text{ units}\right)}{\left(40\;\text{cm}/\text{M}\times \text{mg protein}\right)}\times \text{df}, \end{array}$$

where df = dilution factor and Abs = absorbance.

#### 2.15.7. Superoxide Dismutase (SOD) Activity

The measurement of total SOD activity was performed according to the Misra and Fridovich method with some slight modifications [36]. The principle of this method is based on the inhibition of epinephrine autoxidation. Distilled water (0.2 mL) and 2.5 mL sodium carbonate buffer 0.05 M, pH 10.2 were added to the 0.3 mL buffered epinephrine to initiate the reaction. The absorbance at 480 nm was read for 150 s at 30 s intervals against a blank made up of 2.5 mL buffer, 0.3 mL epinephrine, and 0.2 mL distilled water. The following equation allowed the calculation of the SOD activity:

(3) $$\begin{array}{c} \text{SOD}\left(\;\frac{\text{unit}}{\text{mg protein}}\;\right)=\frac{\text{SOD}\left(\text{units/mL/min}\right)}{\text{protein}\left(\text{mg}/\text{mL}\right)}\times \text{df}, \end{array}$$

where df = dilution factor.

The SOD activity was thereafter expressed as unit/min/mg of protein (UI/mg Prot.)

### 2.16. Statistical Analysis

The results were presented as mean ± SD of triplicate assays. Analyses of data was conducted using one-way ANOVA (analysis of variance) followed by Kruskal-Wallis test and Dunnett's multiple test (SPSS program version 18.0 for Windows, IBM Corporation, New York, NY, USA). The Log probit was used to determinate the IC_50_ using the software XLstat version 7 (Addinsoft, New York, NY, USA) were used to achieve the Pearson Correlation Analysis (PCA). The differences were considered as significant at *p* < 0.05.

## 3. Results

### 3.1. Total Phenol, Flavonoid, and Flavonol Contents of* T. tetraptera *Extract

The total phenol flavonoid and flavonol contents of* T. tetraptera* bark and fruit extracts are represented in Table 1. This result shows that, among the extracts studied, the extract CFH exhibited significant (*p* < 0.05) and highest level of total phenols (37.24 ± 2.00 CAE/g dried extract); flavonoids (11.36 ± 1.88 QE/g dried extract); and flavonols (3.95 ± 0.39 QE/g dried extract).

### 3.2. The HPLC Analysis of the Polyphenols from the Barks and Fruits of* T. tetraptera*

**Figure 1:**
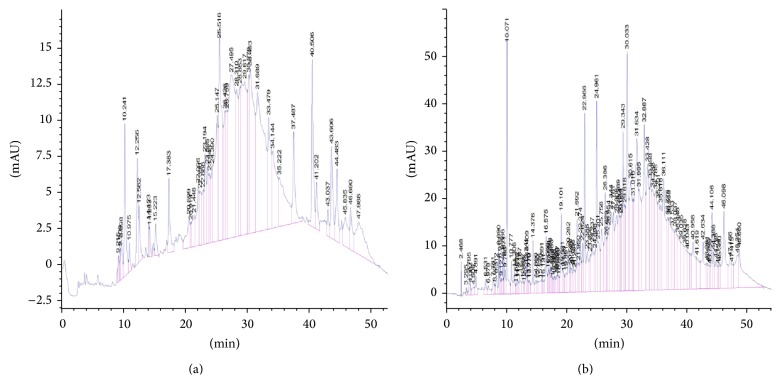
HPLC chromatograms of phenolic extracts from* T. tetraptera* recorded at 280 nm; (a) barks; (b) fruits (TR: 19.10: 3,4-OH benzoic acid; 33.49: apigenin; 25.67: caffeic acid; 23.48: catechin; 29.43: eugenol; 14.38; gallic acid; 25.11: O-coumaric; 21.91: OH-tyrosol; 30.52: P-coumaric acid. 42.19: quercetin; 29.45: rutin; 25.55: syringic acid; 17.35: theobromine; 21.77: tyrosol and 25.27: vanillic acid).

The HPLC profile of the polyphenols from bark and fruit extracts of* T. tetraptera* is represented in Figures 1(a) and 1(b) and Table 2. These results demonstrated that polyphenols molecules are more concentrated in the fruits than the barks. The most represented phenolic compounds such as eugenol, quercetin, rutin, and tyrosol are 5.88–10.79-fold more concentrated in the fruits than the barks (Table 2).

### 3.3. Scavenging Effects of* T. tetraptera *on the DPPH, OH, NO, and ABTS Radicals

The scavenging effects of the* T. tetraptera* extracts on the DPPH, OH, NO, and ABTS radicals are represented in Table 3. These results showed that the sample CFH has the highest free radical scavenging potential with lowest values of IC_50_. These include 31.06 ± 1.57 *μ*g/mL for the DPPH radical, 69.11 ± 1.33 *μ*g/mL for the OH radical, 80.15 ± 2.22 *μ*g/mL for the NO radical, and 102.15 ± 4.01 *μ*g/mL for the ABTS radical. However, the IC_50_ values of the mentioned extract are higher than those of vitamin C used as standard.

### 3.4. Effects of* T. tetraptera* Extracts on the Reductive Activity

**Figure 2:**
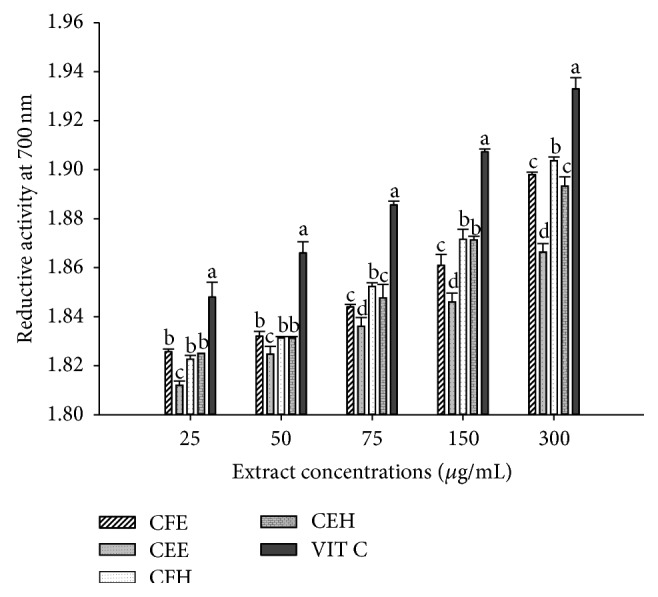
Reductive activity of the different plant extracts. Values are expressed as mean ± SD of three replicates. In the same concentration the values affected with different letter are significantly different at *p* < 0.05. CEH:* T. tetraptera* hydroethanolic extract (bark); CFH:* T. tetraptera* hydroethanolic extract (Fruit); CEE:* T. tetraptera* ethanolic extract (bark); CFE:* T. tetraptera* ethanolic extract (Fruit); VIT C = vitamin C.

The reductive activity of the different extractsof* T. tetraptera* is pictured in Figure 2. The results show that the reductive activity of the extracts increases in the concentration dependent manner. The samples CFE and CFH have the significantly (*p* < 0.05) highest reductive power with values varying from 1.825 ± 0.001 (CFE) and 1.823 ± 0.001 (CFH) to 1.899 ± 0.000 (CFE) and 1.905 ± 0.001 (CFH) at 25 *μ*g/mL and 300 *μ*g/mL, respectively. The vitamin C used as standard showed the significantly (*p* < 0.05) highest reductive activity at all the tested concentrations.

### 3.5. Effects of* T. tetraptera* Extracts on the FRAP Antioxidant Potential

**Figure 3:**
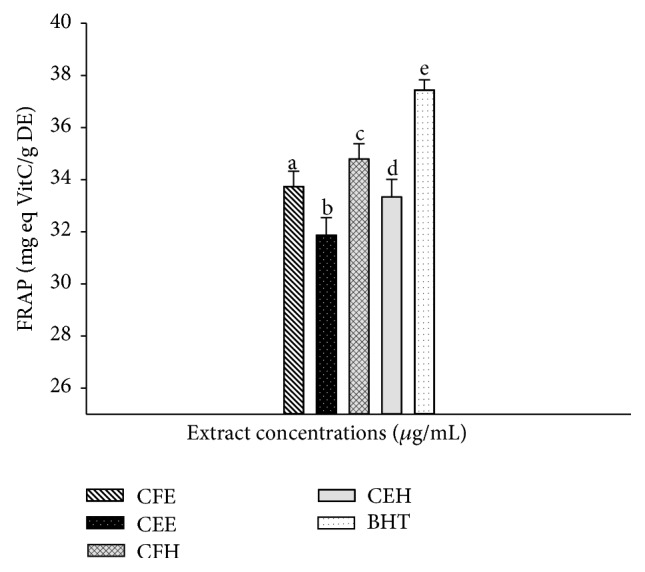
FRAP antioxidant activities of the different plant extracts. Values are expressed as mean ± SD of three replicates. In the same concentration the values affected with different letter are significantly different at *p* < 0.05; CEH:* T. tetraptera* hydroethanolic extract (bark); CFH:* T. tetraptera* hydroethanolic extract (fruit); CEE:* T. tetraptera* ethanolic extract (bark); CFE:* T. tetraptera* ethanolic extract (fruit);* BHT: butylated hydroxytoluene*.

The FRAP antioxidant potential of the different extracts of* T. tetraptera* is represented in Figure 3. This figure shows that the extract CFH has the highest value of FRAP (35.40 ± 0.01 mg eq VitC/g DE) compared to the other extracts demonstrating that extract exhibited the significant antioxidant property. The vitamin C showed the overall significantly (*p* < 0.05) highest FRAP antioxidant potential.

### 3.6. Effects of* T. tetraptera* on the Phosphomolybdenum Antioxidant Power (PAP)

**Figure 4:**
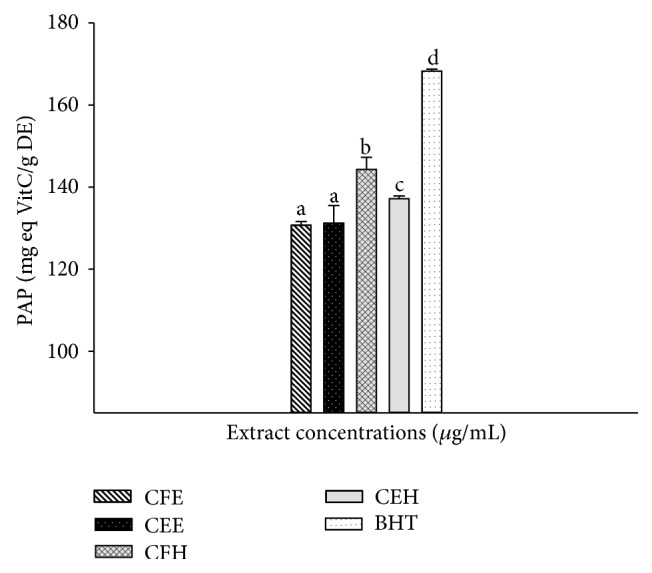
Phosphomolybdenum antioxidant power (PAP) of the different plant extracts. Values are expressed as mean ± SD of three replicates. In the same concentration the values affected with different letter are significantly different at *p* < 0.05 CEH:* T. tetraptera* hydroethanolic extract (bark); CFH:* T. tetraptera* hydroethanolic extract (fruit); CEE:* T. tetraptera* ethanolic extract (bark); CFE:* T. tetraptera* ethanolic extract (fruit);* BHT: butylated hydroxytoluene*.

The results of the phosphomolybdenum antioxidant power (PAP) value of the different extract of* T. tetraptera* are represented in Figure 4 showing that the extract CFH (146.61 ± 0.01 mg eq VitC/g DE) has the significantly (*p* < 0.05) highest PAP value among the tested extracts. The BHT used as standard (170.61 ± 0.01 mg eq VitC/g DE) showed the overall significantly (*p* < 0.05) highest PAP of all the samples.

### 3.7. Effects of* T. tetraptera* Extracts on Lipid Peroxidation

**Figure 5:**
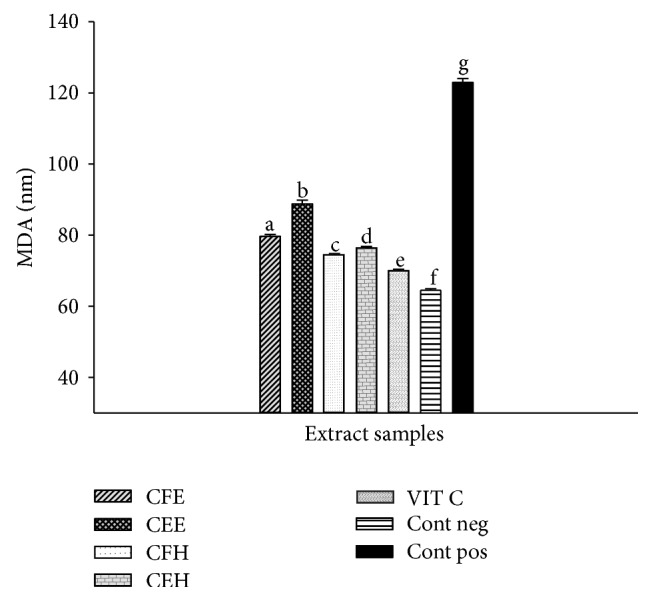
Protective properties of plant extracts against lipid peroxidation. Values are expressed as mean ± SD of three replicates. The values affected with different letter are significantly different at *p* < 0.05; CEH:* T. tetraptera* hydroethanolic extract (bark); CFH:* T. tetraptera* hydroethanolic extract (fruit); CEE:* T. tetraptera* ethanolic extract (bark); CFE:* T. tetraptera* ethanolic extract (fruit); VIT C = vitamin C; Pos. control: oxidant (positive) control. Neg. control: normal (negative) control.

The protective effect of* T. tetraptera* extracts against lipid peroxidation is represented in Figure 5. This figure shows a significantly (*p* < 0.05) higher MDA level in the oxidant (positive) control (123.09 ± 1.08 nM) compared to the normal (negative) control (64.22 ± 0.11 nM). Among the tested samples a significant (*p* < 0.05) decrease of the MDA level was observed with the extract CFH (74.42 ± 2.11 nM) which exhibited the lower MDA concentration compared to the other extracts.

### 3.8. Effects of* T. tetraptera* Extracts on SOD Activity

**Figure 6:**
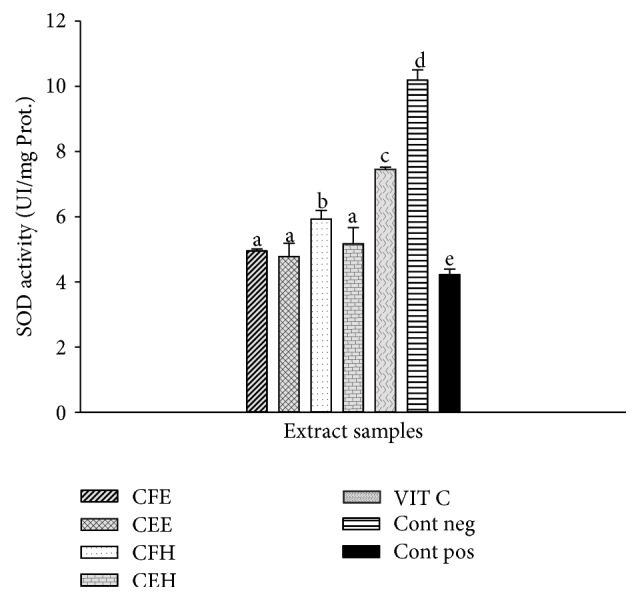
Protective properties of plant extracts on SOD activity. Values are expressed as mean ± SD of three replicates. The values affected with different letter are significantly different at *p* < 0.05; CEH:* T. tetraptera* hydroethanolic extract (bark); CFH:* T. tetraptera* hydroethanolic extract (fruit); CEE:* T. tetraptera* ethanolic extract (bark); CFE:* T. tetraptera* ethanolic extract (fruit); VIT C = vitamin C; Pos. control: oxidant (positive) control. Neg. control: normal (negative) control.

The effects of* T. tetraptera* extracts on SOD activity are represented in Figure 6 showing a significantly (*p* < 0.05) lower SOD activity in the oxidant (positive) control (4.02 ± 0.02 UI/mg Prot.) compared to the negative control (10.11 ± 0.07 UI/mg Prot.). Among the tested samples a significant (*p* < 0.05) increase of the SOD activity of CFH extract was observed (6.21 ± 0.02 UI/mg Prot.) demonstrating that this extract exhibited the higher protective effects on SOD enzyme compared to the other extracts.

### 3.9. Effects of* T. tetraptera* Extracts on Catalase Activity

**Figure 7:**
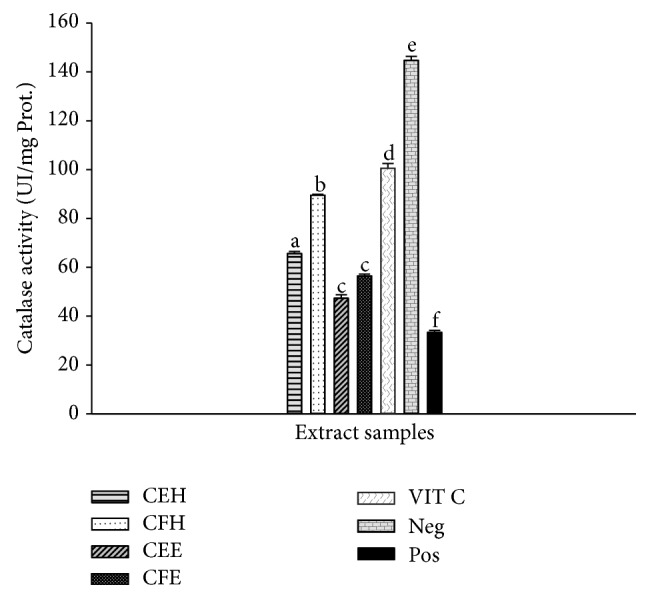
Protective properties of plant extracts on catalase activity. Values are expressed as mean ± SD of three replicates. The values affected with different letter are significantly different at *p* < 0.05; CEH:* T. tetraptera* hydroethanolic extract (bark); CFH:* T. tetraptera* hydroethanolic extract (fruit); CEE:* T. tetraptera* ethanolic extract (bark); CFE:* T. tetraptera* ethanolic extract (fruit); VIT C = vitamin C; Pos. control: oxidant (positive) control. Neg. control: normal (negative) control.

The protective effects of* T. tetraptera* extracts on catalase activity are represented in Figure 7. This figure shows a significantly (*p* < 0.05) lower catalase activity in the oxidant (positive) control (33.53 ± 3.00 UI/mg Prot.) compared to the negative control (145.35 ± 4.98 UI/mg Prot.). A significant (*p* < 0.05) increase of the catalase activity was found with the extract CFH (89.71 ± 3.81 UI/mg Prot.) which exhibited the higher catalase activity compared to the other extracts.

### 3.10. Effects of* T. tetraptera* Extracts on Peroxidase Activity

**Figure 8:**
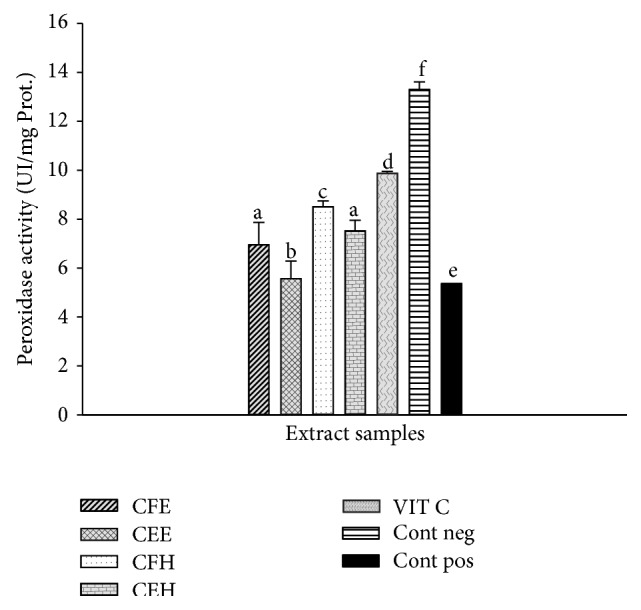
Protective properties of plant extracts: peroxidase activity. Values are expressed as mean ± SD of three replicates. The values affected with different letter are significantly different at *p* < 0.05 CEH:* T. tetraptera* hydroethanolic extract (bark); CFH:* T. tetraptera* hydroethanolic extract (fruit); CEE:* T. tetraptera* ethanolic extract (bark); CFE:* T. tetraptera* ethanolic extract (fruit); VIT C = vitamin C; Pos. control: oxidant (positive) control. Neg. control: normal (negative) control.

The effects of* T. tetraptera* extracts on peroxidase activity are represented in Figure 8. This figure shows that the positive control has the lower peroxidase activity (5.36 ± 0.10 UI/mg Prot.) compared to the negative control (13.30 ± 0.71 UI/mg Prot.). Among the tested samples a significant (*p* < 0.05) increase of the peroxidase activity was observed with the extract CFH (8.52 ± 1.00 UI/mg Prot.) which exhibited the higher peroxidase activity compared to the other extracts.

### 3.11. Correlation and Principal Component Analysis (ACP)

**Figure 9:**
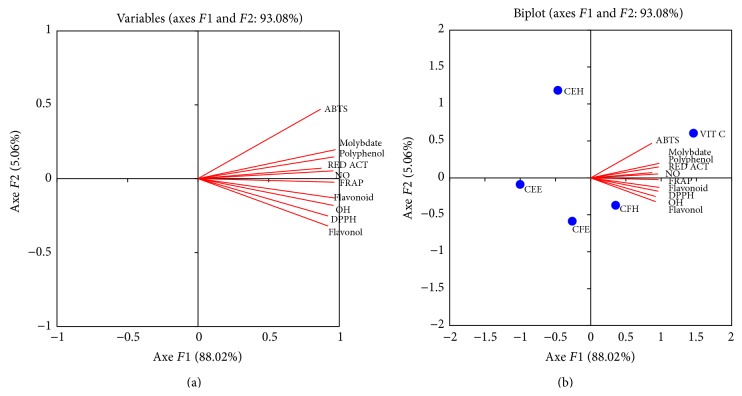
Principal component analysis results on* F*1 ×* F*2 axis of antioxidant and scavenging activities of the extracts tested. Values are expressed as mean ± SD of three replicates. CEH:* T. tetraptera* hydroethanolic extract (bark); CFH:* T. tetraptera* hydroethanolic extract (fruit); CEE:* T. tetraptera* ethanolic extract (bark); CFE:* T. tetraptera* ethanolic extract (fruit); MOLYBDAT: phosphomolybdenum test; flavonol: flavonol assay; polyphenol: polyphenol assay; flavonoid: flavonoid assay; NO: NO radical scavenging test; ABTS: ABTS radical scavenging test; DPPH: DPPH radical scavenging test; OH: OH radical scavenging test; RED ACT: reductive activity test.

**Figure 10:**
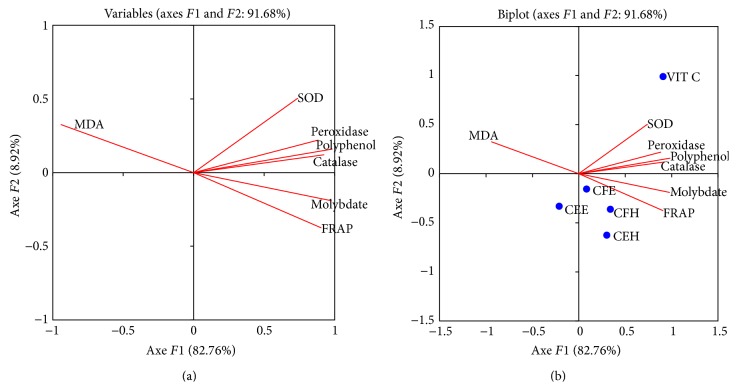
Principal component analysis results on* F*1 ×* F*2 axis of* in vitro* antioxidant assays on rat liver enzymes for the tested extracts. Values are expressed as mean ± SD of three replicates. CEH:* T. tetraptera* hydroethanolic extract (bark); CFH:* T. tetraptera* hydroethanolic extract (fruit); CEE:* T. tetraptera* ethanolic extract (bark); CFE:* T. tetraptera* ethanolic extract (fruit); MOLYBDAT: phosphomolybdenum test; SOD: SOD activity test; catalase: catalase activity test; peroxidase: peroxidase activity test; flavonols: flavonol assay; polyphenol: polyphenol assay; flavonoids: flavonoid assay; FRAP: FRAP antioxidant test; INHIB MDA: MDA inhibition percentage.

The correlation, principal component analysis of the antioxidant assays of the different extracts are presented in Figures 9 and 10 and Tables 4 and 5. The different samples and the antioxidant tests have been projected in a single system; according to the ACP analysis, 93.08% of the variation exists in this system with a contribution of 88.02% for the axis F1 and 5.06 for the axis F2.

Figure 9(a) shows that the total phenolic content is highly and positively correlated with the reductive activity and FRAP assays. The correlation analysis (Table 4) supports this observation by displaying a significant and positive correlation between the total phenolic content, the reductive activity (88.6%), and the FRAP test (92.8%).  Figure 9(b) showed that the extract CFH was closer to the F1 axe than the other tested extracts and therefore could be the most potent extract.

The different extracts and the protective activity assays have been projected in the second system; the ACP analysis explains that 91.68% of the variation exists in this system with a contribution of 82.76% for the axis F1 and 8.92% for the axis F2.  Figure 10(a) showed that the polyphenol test has a higher affinity for the SOD, catalase, and peroxidase tests. A significant (*p* < 0.05) positive correlation between the polyphenol level and the SOD, catalase, and peroxidase tests was observed in the correlation analysis (Table 5).  Figure 10(b) showed a higher affinity for the extract CFH for the different protective tests.

## 4. Discussion and Conclusion

Free radicals are consistently formed in the human body, generally as reactive oxidant species (ROS). They are derived either from normal essential metabolic processes in the human body as by-products of aerobic metabolism or from external sources such as exposure to air pollutants and industrial chemicals such as metal ions [37]. Metals cause severe toxic and deleterious effects on human health by producing various kinds of metabolic disorders and even chronic diseases [37]. The toxicity of ions may be mediated by the induction of oxidative stress which causes damage to cellular components, particularly DNA, and interference with DNA repair systems, resulting in genomic instability and leading to degenerative disorder [38]. Dietary antioxidants are believed to scavenge and/or inhibit the production of free radicals in the body. Plant phytochemicals have been demonstrated to possess higher antioxidant potential [39]. In the present study we investigated the antioxidant potential and free radical scavenging potential of* T. tetraptera* extracts and their protective potential against oxidative mediated ion toxicity.

Numerous methods have been developed to evaluate antioxidant of samples. The DPPH radical has an odd electron, which is responsible for the deep purple color [40]. When DPPH accepts an electron donated by an antioxidant, it is decolorized absorbance indicating that the odd electron of DPPH radical is paired with hydrogen from the antioxidant to form the reduced DPPH-H [40]. The color changes can be quantitatively measured at 517 nm. The results of this study indicate that all the extracts tested can provide hydrogen to DPPH; thus, they have antioxidant properties. Among them, extract CFH possesses the highest DPPH scavenging activity with an IC_50_ of 31.06 ± 1.57 *μ*g/mL. All the extracts from* T. tetraptera* may act as antioxidant by scavenging free radicals. This result corroborates previous studies which demonstrated that plant phenolic compounds could act as radical chelator [41].

Nitric oxide (NO^•^) is a reactive radical that acts as an oxidative biological signaling molecule in diverse physiological processes, including neurotransmission, blood pressure regulation, defense mechanisms, and immune regulation [9]. Nitrosative stress is overproduction of reactive nitrogen species; this may lead to oxidative stress. The tested extracts showed an inhibiting potential on NO radical in a concentration dependent manner. This activity suggests that* T. tetraptera* extracts may inhibit the formation of peroxynitrite and nitric dioxide formed from the reactions of the NO radical* in vivo* [9, 13].

The hydroxyl radical (^•^OH) has a high reactivity, making it the most dangerous radical with a very short* in vivo* half-life of approximately, 10^−9^ s [42]. The ^•^OH radical could mediate damage to cell structures, including lipids and membranes, proteins and nucleic acids. Our results showed a reductive power capacity of the extracts tested which was proportional with the extract concentration. The extract CFH exhibited the higher power reductive potential and can serve as free radical inhibitor such as ABTS. This activity may be linked to its higher concentration in flavonoids such as quercetin which was found to be in a high concentration in* T. tetraptera* fruit using HPLC. Several mechanisms of antioxidant properties of phenolic molecules have been identified such as electron donor through the reduction of Fe^3+^ complex to its Fe^2+^, inhibitor of the Fenton reaction by chelating the ion molecules. Our results demonstrated that* T. tetraptera* extracts exert their antioxidant activities using all these mechanisms (Figures 4 and 5). Previous studies have demonstrated those plants based antioxidants which possess polyphenol content usually used these mechanisms [8, 39]. Moreover the ACP correlation analysis showed a significant (*p* < 0.05) and positive correlation between the total phenol content and the reductive power (Figure 10(b) and Table 4).

In the FRAP assay antioxidants in the sample reduce ferric-tripyridyltriazine complex (Fe^3+^-TPTZ) in stoichiometric excess to a blue ferrous form (Fe^2+^) with an increase in absorbance at 593 nm [43]. The present study revealed that the tested samples can reduce Fe^3+^-TPTZ to its ferrous form; therefore, they possess higher potential antioxidant capacity. The* T. tetraptera* extracts could inhibit the radical damages that are mediated by ions. This result corroborates previous research which demonstrated that plant polyphenols are powerful antioxidants [43, 44]. Phosphomolybdate antioxidant power (PAP) is another assay that is performed to assess the overall antioxidant activity of the extract. In the presence of antioxidant molecule in the sample, reduce Mo (VI) to Mo (V) with subsequent formation of green colored phosphomolybdenum V complex exhibiting maximum absorbance at 700 nm [13]. The tested extracts in this study presented a higher PAP.

Lipid peroxidation is a metabolic process under normal aerobic conditions and is one of the highly investigated consequences of ROS action on membrane structure and function [33]. Polyunsaturated fatty acids (PUFA), the main components of membrane lipids, are susceptible to peroxidation. OH can react with the methylene groups of PUFA forming conjugated dienes, lipid peroxy radicals (LOOP), and hydroperoxides (LOOH) [33]. The lipid hydroperoxides produced can undergo reductive cleavage by reduced metals such as Fe^2+^ and produce lipid alkoxyl radical which can initiate additional chain reactions [33]. The final stable products of peroxidation are aldehydes which react with thiobarbituric acid (TBA) to form TBA-malonaldehyde adduct with an absorbance maximum at 532 nm [4, 13]. In the present study,* T. tetraptera* extracts showed a higher inhibition of the lipid peroxidation which characterized a decrease of the level of MDA. These protective effects of the lipid peroxidation varied depending on the part of the plants, the solvent used for its extraction, and the quality and the quantity of phytochemical compounds found in the extract [13]. The extract CFH showed the highest inhibitory effects of MDA. This activity could be attributed to the phenolic content of the sample. Indeed the CFH sample has the highest phenolic and flavonoid content between the tested samples. Previous study demonstrated that phenols are good preventers of lipid peroxidation and may be used against deleterious effects of the ROS on cell membrane [10, 13]. In the same way, it has been shown that quercetin may protect mouse erythrocytes from iron-mediated lipid peroxidation by binding iron [45].

The human body has evolved highly complex enzymatic antioxidant systems which protect the cells and organ against free radical damage [9]. This system includes enzymatic antioxidants such as glutathione peroxidase, catalase, and superoxide dismutase (SOD). The kinetic mechanism reaction of the Fenton-like reagent (Fe(III)/H_2_O_2_) has been as well as its efficiency for preventing or removing pollutants [46]. Fenton's oxidation appeared to be the most promising method, in terms of cost-effectiveness and ease of operation [46]. The investigation of the effects of* T. tetraptera* extracts on the protection of these enzyme activities in presence of ion was realized. The results demonstrated that these extracts protected these enzymes from oxidative deterioration. This is confirmed through the increasing of enzymes activities of SOD, CAT, and glutathione peroxidase compared to the positive control (Figures 7, 8, and 9). Indeed it has been shown that polyphenols have many different biological activities, among them are enzyme regulation and antioxidant behavior [45]. These results corroborate those obtained by previous research demonstrating the ability of polyphenols to chelate and remove iron from iron-loaded hepatocytes [45]. Moreover a significant positive correlation has been demonstrated by ACP correlation analysis between the total phenol level and the increase of SOD, catalase, and peroxidase activities. The HPLC profile of* T. tetraptera* showed that extract from the fruits is qualitatively and quantitatively more concentrated in phenolic compound than the barks. This result may justify the higher antioxidant and protective potential of the sample CFH.

In summary,* T. tetraptera* extracts possess various and diversified phenolic compounds with free radical scavenging activities, antioxidant properties, and a high protective potential against oxidative mediated ion toxicity. Nevertheless, more studies are required in order to set it as a potential drug against metal toxicity, identify the active molecule, and investigate its mechanism of action using an appropriate dose.

## Figures and Tables

**Table 1:** Total phenol, flavonoid, and flavonol contents of the plant extracts.

Samples	Phenolic composition		
	Total phenols (CAE/g dried extract)	Flavonoids (QE/g dried extract)	Flavonols (QE/g dried extract)
CEH	30.56 ± 1.06^a^	6.31 ± 1.00^a^	1.56 ± 0.01^a^
CFH	37.24 ± 2.00^b^	11.36 ± 1.88^b^	3.95 ± 0.39^b^
CEE	26.65 ± 0.01^c^	5.22 ± 1.12^a^	1.55 ± 0.02^a^
CFE	29.46 ± 1.19^d^	10.38 ± 1.77^b^	3.90 ± 0.57^b^

Values are expressed as mean ± SD of three replicates. In the same concentration the values affected with different letter are significantly different at *p* < 0.05; CEH: *T. tetraptera* hydroethanolic extract (bark); CFH:* T. tetraptera* hydroethanolic extract (fruit); CEE:* T. tetraptera* ethanolic extract (bark); CFE:* T. tetraptera* ethanolic extract (fruit); CAE: caffeic acid equivalent; QE: quercetin.

**Table 2:** Representation of the level of phenolic compounds in the different plant parts.

Phenol standards	Standard retention time	*T. tetraptera *(fruits)		*T. tetraptera *(barks)	
Characteristics	T.R (Decker #547)	*A* (mUA)	Conc. (mg/g DW)	*A* (mUA)	Conc. (mg/g DW)
3.4-OH benzoic acid	19.10 ± 00	00 ± 00	00 ± 00	226.00 ± 00	8.91 ± 00
Apigenin	33.49 ± 00	39.16 ± 00	0.01 ± 00	841.09 ± 00	0.21 ± 00
Caffeic acid	25.67 ± 00	1210.83 ± 00	22.72 ± 00	38.34 ± 00	0.72 ± 00
Catechin	23.48 ± 00	785.99 ± 00	57.13 ± 00	223.34 ± 00	16.23 ± 00
Eugenol	29.43 ± 00	3316.70 ± 00	958.81 ± 00	564.46 ± 00	163.18 ± 00
Gallic acid	14.38 ± 00	383.80 ± 00	9.84 ± 00	148.70 ± 00	3.81 ± 00
O-Coumaric acid	25.11 ± 00	1357.50 ± 00	41.91 ± 00	487.81 ± 00	15.06 ± 00
OH-tyrosol	21.91 ± 00	737.50 ± 00	68.13 ± 00	88.6 ± 00	8.18 ± 00
P-Coumaric acid	30.52 ± 00	2595.24 ± 00	50.82 ± 00	1078.95 ± 00	21.13 ± 00
Quercetin	42.19 ± 00	3765.61 ± 00	353.78 ± 00	360.40 ± 00	33.86 ± 00
Rutin	29.45 ± 00	2500.70 ± 00	210.54 ± 00	231.55 ± 00	19.50 ± 00
Syringic acid	25.55 ± 00	1210.83 ± 00	30.02 ± 00	00 ± 00	00 ± 00
Theobromine	17.35 ± 00	643.97 ± 00	21.59 ± 00	74.21 ± 00	2.49 ± 00
Tyrosol	21.77 ± 00	2072.02 ± 00	121.05 ± 00	215.35 ± 00	12.58 ± 00
Vanillic acid	25.27 ± 00	1129.18 ± 00	36.07 ± 00	266.50 ± 00	8.51 ± 00

Conc.: concentration; DW: dried weight; T.R: retention time; *A*: area.

**Table 3:** Different values of IC_50_ of the plant extracts on the different radicals tested.

Samples tests	IC_50_ (*μ*g/mL)			
	DPPH	OH	NO	ABTS
CEH	84.60 ± 0.07^a^	96.07 ± 2.01^a^	195.18 ± 3.01^a^	132.34 ± 2.36^a^
CFH	31.06 ± 1.57^b^	69.11 ± 1.33^b^	80.15 ± 2.22^b^	102.15 ± 4.01^b^
CEE	99.37 ± 2.66^c^	102.72 ± 3.02^c^	131.88 ± 2.99^c^	178.54 ± 2.99^c^
CFE	153.12 ± 2.22^d^	108.69 ± 2.00^d^	137.26 ± 2.11^d^	168.34 ± 2.43^d^
VIT C	2.55 ± 1.31^e^	27.76 ± 1.66^e^	55.84 ± 2.96^e^	68.17 ± 2.12^e^

Values are expressed as mean ± SD of three replicates. In the same concentration the values affected with different letter are significantly different at *p* < 0.05; CEH: *T. tetraptera* hydroethanolic extract (bark); CFH:* T. tetraptera* hydroethanolic extract (fruit); CEE:* T. tetraptera* ethanolic extract (bark); CFE:* T. tetraptera* ethanolic extract (fruit); VIT C = vitamin C.

**Table 4:** Correlation between free radicals scavenging properties and the determination of polyphenols compounds.

	DPPH	OH	NO	ABTS	RED ACT	FRAP	Molybdate	Polyphenol	Flavonoid	Flavonol
DPPH	1									
OH	0.955^*∗*^	1								
NO	0.850^*∗*^	0.787	1							
ABTS	0.676	0.769	0.745	1						
RED ACT	0.843^*∗*^	0.932^*∗*^	0.742	0.887^*∗*^	1					
FRAP	0.876^*∗*^	0.920^*∗*^	0.777	0.822^*∗*^	0.947^*∗*^	1				
Molybdate	0.861^*∗*^	0.902^*∗*^	0.869^*∗*^	0.927^*∗*^	0.926^*∗*^	0.929^*∗*^	1			
Polyphenol	0.829^*∗*^	0.865^*∗*^	0.876^*∗*^	0.879^*∗*^	0.886^*∗*^	0.928^*∗*^	0.977^*∗*^	1		
Flavonoid	0.876^*∗*^	0.931^*∗*^	0.817^*∗*^	0.780	0.923^*∗*^	0.943^*∗*^	0.915^*∗*^	0.942^*∗*^	1	
Flavonol	0.871^*∗*^	0.921^*∗*^	0.750	0.656	0.877^*∗*^	0.894^*∗*^	0.822^*∗*^	0.854^*∗*^	0.978^*∗*^	1

^**∗**^Significant values *p* = 0.050 (bilateral test).

Molybdate: phosphomolybdenum test; flavonols: flavonol assay; polyphenol: polyphenol assay; flavonoids: flavonoid assay; NO: NO radical scavenging test; ABTS: ABTS radical scavenging test; DPPH: DPPH radical scavenging test; OH: OH radical scavenging test; RED ACT: reductive activity test.

**Table 5:** Correlation analysis results of *T. tetraptera *extracts demonstrated by Pearson's coefficients for *in vitro* antioxidant assays on rat liver enzymes.

	Peroxidase	Catalase	SOD	MDA	FRAP	Molybdate	Polyphenol
Peroxidase	1						
Catalase	0.950^*∗*^	1					
SOD	0.592	0.587	1				
MDA	−0.755	−0.813^*∗*^	−0.545	1			
FRAP	0.642	0.721	0.567	−0.979^*∗*^	1		
Molybdate	0.776	0.847^*∗*^	0.668	−0.984^*∗*^	0.972^*∗*^	1	
Polyphenol	0.882^*∗*^	0.927^*∗*^	0.807^*∗*^	−0.870^*∗*^	0.828^*∗*^	0.930^*∗*^	1

^*∗*^Significant values *p* = 0.050 (bilateral test).

SOD: SOD activity test; catalase: catalase activity test; peroxidase: peroxidase activity test; flavonol: flavonol assay; polyphenol: polyphenol assay; flavonoids: flavonoid assay; FRAP: FRAP antioxidant test; MDA: MDA assay; INHIB MDA: MDA inhibition percentage.

## Abbreviations

DPPH: 2,2-Diphenyl-1-picrylhydrazyl
FRAP: Ferric reducing ability of plasma
Vit C: Vitamin C
ABTS: 2,2-Azinobis(3-ethylbenzthiazoline)-6-sulfonic acid
BHT: Butylated hydroxytoluene
RNS: Reactive nitrogen species
ROS: Reactive oxygen species
FRAP: Ferric reducing antioxidant power
MDA: Malondialdehyde
TBA: Thiobarbituric acid
H_2_O_2_: Hydrogen peroxide.
